# Measuring aortic pulse wave velocity using high-field cardiovascular magnetic resonance: comparison of techniques

**DOI:** 10.1186/1532-429X-12-26

**Published:** 2010-05-11

**Authors:** El-Sayed H Ibrahim, Kevin R Johnson, Alan B Miller, Jean M Shaffer, Richard D White

**Affiliations:** 1 Department of Radiology, University of Florida College of Medicine, Jacksonville, Florida, USA; 2 Department of Medicine, University of Florida College of Medicine, Jacksonville, Florida, USA

## Abstract

**Background:**

The assessment of arterial stiffness is increasingly used for evaluating patients with different cardiovascular diseases as the mechanical properties of major arteries are often altered. Aortic stiffness can be noninvasively estimated by measuring pulse wave velocity (PWV). Several methods have been proposed for measuring PWV using velocity-encoded cardiovascular magnetic resonance (CMR), including transit-time (TT), flow-area (QA), and cross-correlation (XC) methods. However, assessment and comparison of these techniques at high field strength has not yet been performed. In this work, the TT, QA, and XC techniques were clinically tested at 3 Tesla and compared to each other.

**Methods:**

Fifty cardiovascular patients and six volunteers were scanned to acquire the necessary images. The six volunteer scans were performed twice to test inter-scan reproducibility. Patient images were analyzed using the TT, XC, and QA methods to determine PWV. Two observers analyzed the images to determine inter-observer and intra-observer variabilities. The PWV measurements by the three methods were compared to each other to test inter-method variability. To illustrate the importance of PWV using CMR, the degree of aortic stiffness was assessed using PWV and related to LV dysfunction in five patients with diastolic heart failure patients and five matched volunteers.

**Results:**

The inter-observer and intra-observer variability results showed no bias between the different techniques. The TT and XC results were more reproducible than the QA; the mean (SD) inter-observer/intra-observer PWV differences were -0.12(1.3)/-0.04(0.4) for TT, 0.2(1.3)/0.09(0.9) for XC, and 0.6(1.6)/0.2(1.4) m/s for QA methods, respectively. The correlation coefficients (r) for the inter-observer/intra-observer comparisons were 0.94/0.99, 0.88/0.94, and 0.83/0.92 for the TT, XC, and QA methods, respectively. The inter-scan reproducibility results showed low variability between the repeated scans (mean (SD) PWV difference = -0.02(0.4) m/s and r = 0.96). The inter-method variability results showed strong correlation between the TT and XC measurements, but less correlation with QA: r = 0.95, 0.87, and 0.89, and mean (SD) PWV differences = -0.12(1.0), 0.8(1.7), and 0.65(1.6) m/s for TT-XC, TT-QA, and XC-QA, respectively. Finally, in the group of diastolic heart failure patient, PWV was significantly higher (6.3 ± 1.9 m/s) than in volunteers (3.5 ± 1.4 m/s), and the degree of LV diastolic dysfunction showed good correlation with aortic PWV.

**Conclusions:**

In conclusion, while each of the studied methods has its own advantages and disadvantages, at high field strength, the TT and XC methods result in closer and more reproducible aortic PWV measurements, and the associated image processing requires less user interaction, than in the QA method. The choice of the analysis technique depends on the vessel segment geometry and available image quality.

## Background

The aorta sets the pattern for total systemic compliance of the central cardiovascular network. It expands rapidly during systolic contraction of the left ventricle, temporarily accommodating 50% or more of its stroke volume, and then retracts during diastole, helping to maintain distal antegrade arterial flow and downstream organ perfusion, as well as proximal retrograde flow and pressure transmission for aortic valve closure and coronary artery/myocardial perfusion [1,2]. Maintaining the aortic viscoelastic properties is essential for proper cardiovascular physiology [3,4]. In recent years, a great deal of emphasis has been placed on the role of aortic stiffness as an independent contributor to the development of cardiovascular disease [5]. The assessment of arterial stiffness is increasingly used in clinical assessment of patients because the mechanical properties of arteries are altered in a variety of pathological states (e.g. coarctation of the aorta) [1]. Description of these alterations, and the manner by which vessel wall mechanics may influence the development of vascular pathology, would assist in understanding patient's cardiovascular condition and help plan appropriate therapy.

The assessment of aortic compliance has, until recently, required invasive methods and/or technically complicated procedures using pressure catheters [6]. Such approaches are not feasible for studying arterial wall motion and strain in human subjects [7]. Recently, noninvasive measurements have been made possible with Doppler ultrasound and cardiovascular magnetic resonance (CMR) [8-12]. There are several advantages to using CMR over Doppler ultrasound, such as the ability to view the entire vessel regardless of the vessel angle, depth, or acoustic window. Furthermore, reproducibility studies with Doppler ultrasound have revealed susceptibility to transducer placement and angle, factors that affect the apparent path length of the pulse wave [8].

Aortic stiffness can be noninvasively expressed using pulse wave velocity (PWV), which is the rate at which the flow or pressure wave propagates down the vessel [8,13,14]. PWV is directly related to vessel wall elasticity [15,16], and can be estimated by measuring resulting changes in flow or vessel diameter [17]. It is vessel wall characteristics, not the path curvature, that primarily determine PWV. The advantage of assessing distensibility from PWV is that it does not require pressure measurement, which is costly and requires specific medical experience. PWV, in general, has previously been validated against pressure catheters in estimating vessel compliance [18]. CMR based approaches to PWV measurement have subsequently been validated in both phantoms [8,11] and in human subjects [19-24].

Several methods have been proposed to determine PWV using velocity-encoded CMR images, including transit-time (TT) [25], flow-area (QA) [23], and cross-correlation (XC) [26] methods. There have been assessments of reproducibility in normals [16,19,26,27] and non-tachycardic, normotensive patients with suspected coronary artery disease using 1.5 Tesla CMR methods [19]. However, the reproducibility and comparison of these different techniques have not yet been studied in a large diverse group of patients for relative durability and reproducibility of the CMR methods at any field strength, moreover at 3 Tesla. In this work, the TT, QA, and XC techniques performed using high field strength CMR are tested on human subjects representing a range of cardiovascular conditions, and compared to each other. Inter-observer, intra-observer, and scan-rescan variabilities are studied, along with the advantages and disadvantages of each technique.

Finally, to illustrate the importance of PWV using CMR, aortic stiffness is assessed in a group of five diastolic heart failure patients by measuring PWV, and the results are related to various parameters of diastolic dysfunction and compared to results from volunteers. Diastolic heart failure is accompanied by an isolated abnormality in diastolic LV function and by reduced aortic compliance [28,29], which may play a significant role by impairing systemic load interaction with diastolic function and elevating cardiac metabolic needs under stress [30]. Thus, one factor that may contribute to diastolic heart failure pathophysiology is the abnormal ventricular-arterial interaction because of stiffening of both systems. This factor may result in increased cardiac energy needed to supply blood flow [30].

## Methods

### Study population

Fifty consecutive cardiac patients referred to our imaging center, along with six normal volunteers, were scanned on a 3 Tesla CMR system (Magnetom TIM TRIO, Siemens Healthcare, Erlangen, Germany) with electrocardiogram (ECG) gating. The study group was clinically diverse (Table 1) in order to assure relative durability and reproducibility of the tested methods. The patients were referred to our imaging facility due to various heart diseases including myocardial ischemia and viability (n = 25), non-ischemic heart disease (n = 10), ischemic myocardial damage (n = 5), semilunar valve stenosis (n = 4), hypertrophic obstructive cardiomyopathy (n = 4), myocarditis (n = 1), and intracavity mass (n = 1). The patients were asked to participate in the study, which was approved by our Institutional Review Board. All patients and volunteers gave written informed consent.

**Table 1:** Diversity in the study group (mean ± standard deviation (SD))

Parameter	Mean ± SD
Sex = 32 males & 18 females	

Age (years old)	55 ± 17

Heart rate (beats per minute)	69 ± 14

Systolic/diastolic blood pressure (mm Hg)	140 ± 19/78 ± 14

End-diastolic LV volume (ml)	163 ± 55

Ejection fraction (% EDV)	55 ± 13

LV mass (gm)	150 ± 48

### CMR technique

The patients were placed in a supine position and imaged using a twelve-element phased-array coil. After scouting, three series of velocity-encoded images of the aorta were acquired. The first series was acquired in an oblique-sagittal position along the path of the thoracic aorta with in-plane craniaocaudal/head-to-foot velocity encoding. Next, two cross-sectional views of the descending aorta, one at the level of the pulmonary arteries and the other at a level proximal to the renal arteries, were acquired with through-plane velocity encoding. The imaging parameters were as follows: TR = 13 ms, which includes reference and velocity-encoded acquisitions (i.e. two RF pulses with 6.5 ms in-between); TE = 3 ms; true temporal resolution = 13 ms; interpolated reconstruction matrix = 256 × 192; flip angle = 15°; slice thickness = 8 mm; pixel size = 1.17 × 1.17 mm^2^; velocity encoding value (venc) = 150 cm/s (with concomitant gradient correction); number of reconstructed heart phases = 128 with retro-gated acquisition; number of averages = 1; acceleration factor = 2 with separate pre-scan acquisition of reference lines; FOV = 300 mm; percent phase FOV = 75; average scan time = 26 s/slice of shallow breathing (average RR interval of 900 ms is assumed). The six volunteer scans were repeated with new table positioning and plane scouting to test inter-scan reproducibility.

**Figure 1:**
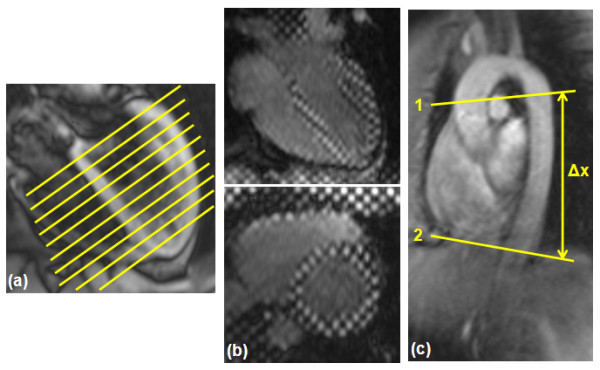
**Acquired CMR images**. Three sets of CMR images are acquired for the diastolic heart failure patients: (a) a stack of parallel short-axis cine images covering the left ventricle from base to apex to measure the filling pattern and myocardial thickness. (b) Four-chamber (up) and short-axis (down) cine tagged images to measure myocardial strain. (c) Two velocity-encoded aortic cross sections, separated by distance Δx to measure the pulse wave velocity.

Additional two sets of images were acquired in the subgroup of five diastolic heart failure patients and five normal volunteers (Figure 1). The first set consisted of a series of cine short-axis (SAX) slices covering the LV from base to apex to calculate the LV blood volume, filling rate, and myocardial thickness. The second set of images consisted of three SAX (basal, mid, and apical) and one four-chamber (4CH) cine tagged images to calculate myocardial strain. The imaging parameters for the cine SAX images were: 2D gradient-echo FLASH sequence; TR/TE = 40/1.2 ms; flip angle = 15°; slice thickness = 8 mm; # cardiac phases = 23; # averages = 1; pixel size = 1.8 × 1.8 mm^2^; bandwidth (BW) = 475 Hz/pixel; scan time = 9 s (breath-hold). The imaging parameters for the tagged images were: TR/TE = 48/4 ms; flip angle = 12°; # cardiac phases = 25; tag separation = 7 mm; slice thickness = 8 mm; # averages = 1; pixel size = 1.5 × 1.5 mm^2^; BW = 185 Hz/pixel; scan time = 13 s (breath-hold).

### Data analysis

**Figure 2:**
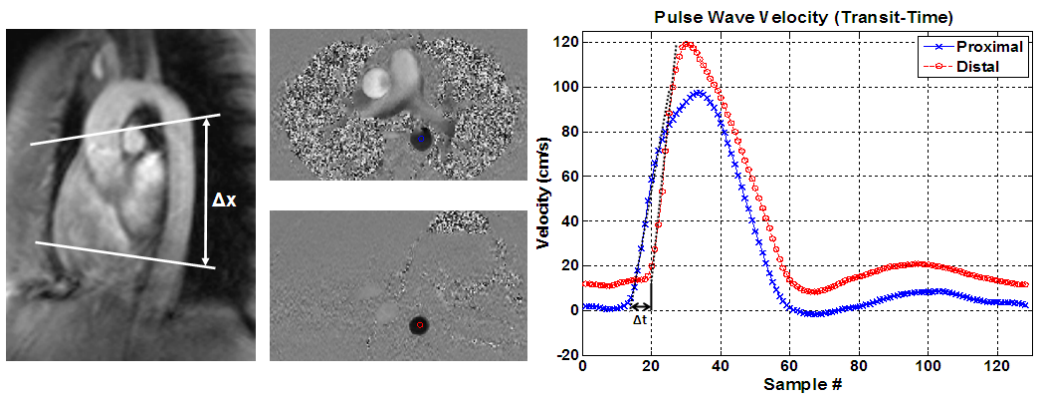
**Transit-time method for calculating PWV**. Velocity curves (right) from a volunteer scan are computed at two distant cross sections (middle) along the descending aorta (left). PWV = Δx/Δt, where Δx is the distance between the two locations and Δt is the time difference between the two velocity curves.

The images were transferred to a personal computer, where they were analyzed using in-house software created with MATLAB (The MathWorks, Inc., Natick, MA) on a 2.4 GHz personal computer. Three software modules were created to implement different PWV analysis techniques: TT, QA, and XC. The time required to analyze each set of images was computed with the help of a stopwatch to compare the efficiency of different techniques. The combination of the computer processing time and manual operator intervention (e.g. manual segmentation in the QA method) was reported. In the TT module, cross-sectional views (magnitude images) of the proximal and distal descending aorta were displayed, and the user was asked to pick a seed point at the center of each vessel cross-section (Figure 2). The program then accessed the sequence of velocity-encoded images to calculate blood flow velocity averaged across a small 5 × 5 pixels region-of-interest (ROI) centered at the seed point. In velocity-encoded images, the pixel gray level is proportional to blood velocity, where the proportionality constant depends on the venc value encoded in the pulse sequence: white and black represent the maximum velocity (equal to the venc value) in the forward and backward directions, respectively. The travelling time (Δ*t*) of the velocity waveform between the two sites was automatically calculated as the time duration between the onset of the curves, determined as the intersection point of the curve upslope and base velocity (Figure 2). Base velocity was defined as the minimum value of the first 10 points of the velocity waveform. The up-steeping edge was identified by the line connecting the points at 20% and 80% of the maximum velocity in the waveform. It should be noted that other approaches have been previously considered for calculating the travelling time in the TT method [31] (e.g. peak systolic velocity, halfway up, or second derivative). PWV was computed as shown in Equation 1, with Δx as the distance along the centerline between the two cross-sections.(1)

**Figure 3:**
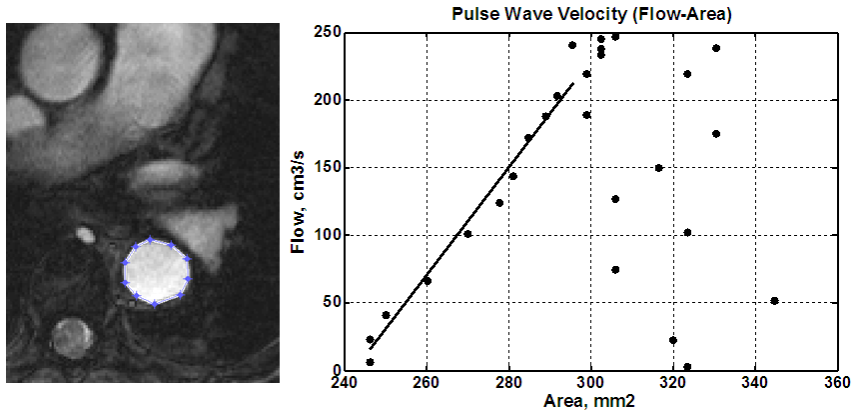
**Flow-area method**. PWV results from a volunteer scan. A cross section of the aorta is shown (left), where the user marks the aorta boundary. The panel on the right shows the change in aortic cross sectional area versus total flow at different frames in the cardiac cycle. A line is fitted to the data during the initial slope of the curve at early systole, from which PWV is calculated. After systole, ROIs were drawn large to separate them from earlier points and avoid confounding the linear fit.

**Figure 4:**
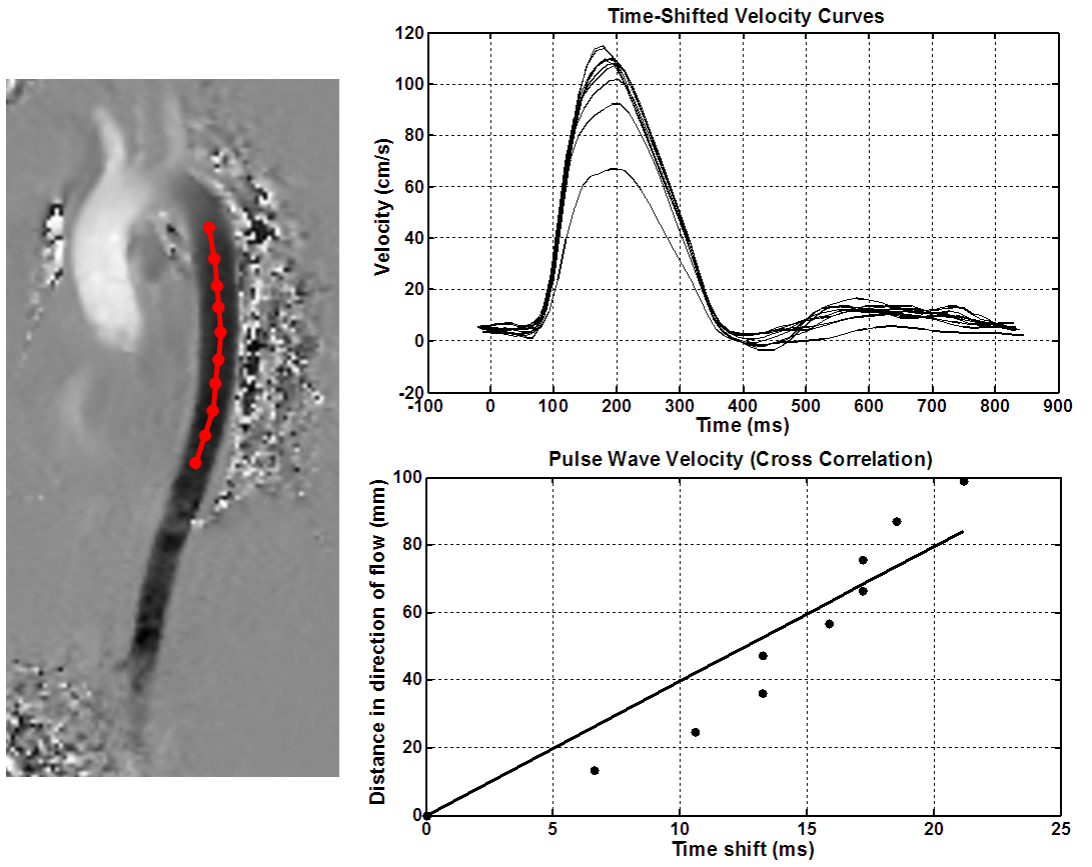
**Cross-correlation method**. Flow patterns (up) from a volunteer scan are computed at several points along the descending aortic path (left). Cross correlation is used to estimate the time shift between consecutive points. Linear least-square fitting is used to calculate PWV (down).

In the QA module, an enlarged view of the aortic cross-section was displayed frame-by-frame during the upslope of the velocity curve (10-15 frames). User-defined ROIs were drawn to define the vessel boundary in each frame (Figure 3), from which the vessel cross-sectional area (A) and blood flow (Q) were calculated, as in Equations 2 and 3:(2)

where *n *is the number of pixels encountered within the vessel boundary; Δ*x *and Δ*y *are the pixel dimensions; and *v*_*i *_is the blood velocity at pixel *i*. The relationship between blood flow and vessel cross-sectional area during early systole was approximated as a first-order linear equation using minimum squared error (Figure 3), and PWV is estimated as in Equation 4:(4)

where Δ*Q *and Δ*A *are the change in total flow and aortic cross-sectional area. Finally, in the XC module, a sagittal view of the aorta was displayed, and the user was asked to select points along the centerline of the aorta (Figure 4). The velocity waveform was calculated at each of the selected points by accessing the velocity-encoded images. Each velocity waveform was then cross-correlated with the waveform at the first point in order to calculate the phase shift between the velocity curves, or travelling time, in milliseconds. It should be noted that, due to its nature, the cross-correlation technique is not affected by varying vertical offsets in the velocity images. The relationship between the travelling times and the distances between the points was approximated as a first-order linear equation using minimum squared error (Figure 4), and the line slope was used as an estimate of PWV. The assumption of zero time-shift at zero distance is used in the line fitting. However, minor variations from this ideal case could be attributed to local differences in vessel wall characteristics across the aortic path, or to the fact that reflection waves were not considered in the analysis tool. It should be also noted that only one-directional (head to foot) velocity encoding was applied in the measuring sequence, which may not be ideal for curved aortic path.

The images were analyzed by two experts experienced in CMR to measure the *inter-observer *variability. One of the experts analyzed the images twice with a two week separation to measure the *intra-observer *variability. He also analyzed the measurements from the volunteers repeated scans to test the *inter-scan *reproducibility.

Despite the lack of gold-standard catheter measurements in this study, the relationship between PWV and pulse pressure (diastolic blood pressure - systolic blood pressure), and LV mass to (end diastolic) volume ratio (mvr) was studied to illustrate the value of PWV as an indicator of different cardiac parameters. To be consistent, this analysis was conducted on the uniform group of ischemic patient (n = 25). LV mvr was used as it reflects the relationship between the chamber mass and volume, as previously described [32]. For this group of patients, the correlation coefficients were calculated between PWV and pulse pressure, and between PWV and mvr. In addition, the mean PWV was calculated, and the average pulse pressure and mvr values were calculated for the sub-groups with PWV above and below the mean value. Finally, the correlation coefficient between PWV and patient age was calculated for the whole study group.

The cine SAX images were transferred to a multi-modality workstation where they were semi-automatically analyzed (Argus software, Siemens Medical Solutions, USA) to determine LV blood volume, filling rate, and end-diastolic myocardial thickness. The tagged images were analyzed using the harmonic phase (HARP) [33] tool in Diagnosoft software to determine myocardial strain.

### Statistics

Finally, the measurements from the different techniques were compared to each other: *TT-XC*, *TT-QA*, and *XC-QA*. Regression analysis was conducted between different methods, and measurement agreement was evaluated using the 95% limits-of-agreement approach proposed by Bland and Altman [34]. The differences significance was tested using paired *t*-test with p < 0.05 considered statistically significant.

## Results

**Figure 5:**
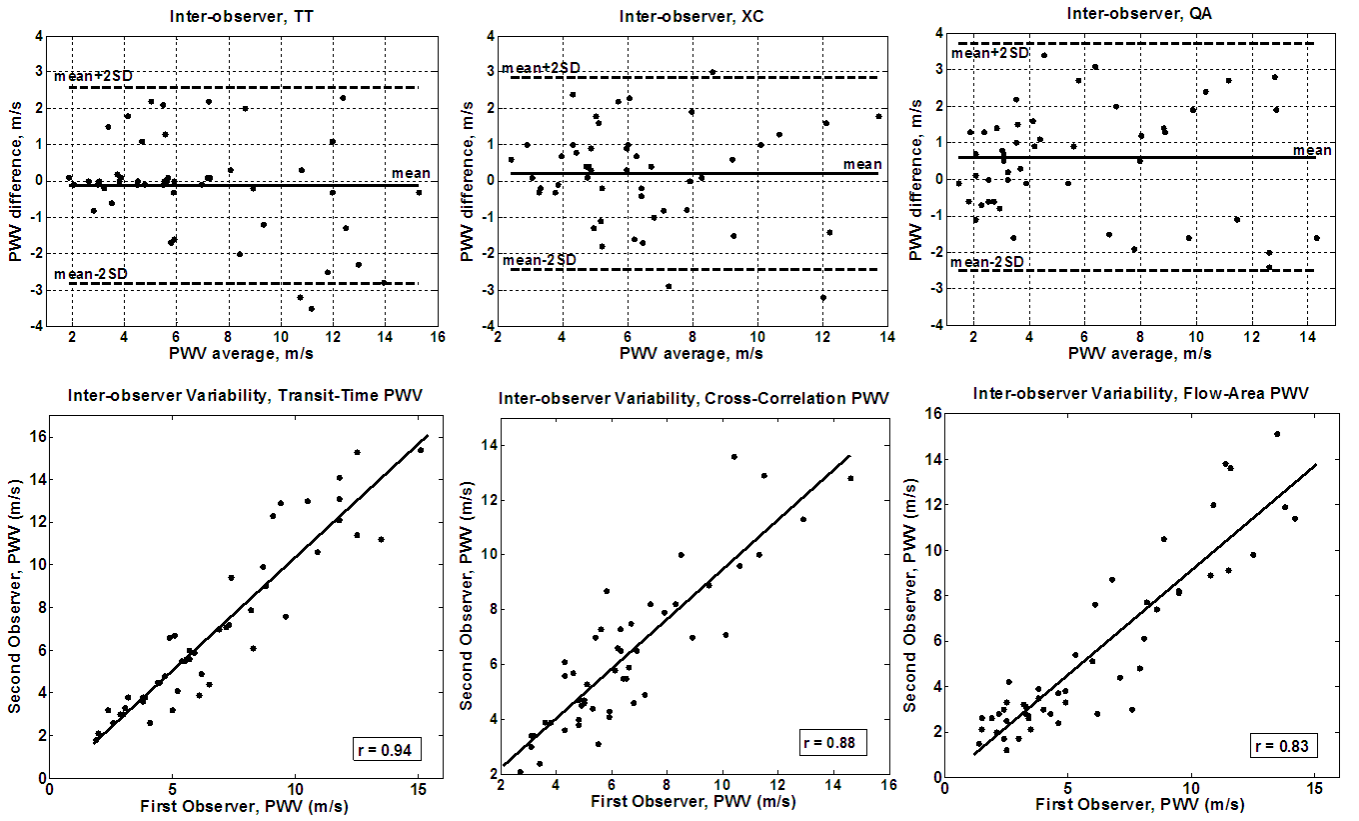
**Inter-observer variability**. Bland-Altman (up) and regression analysis (down) are shown for transit-time (left), cross-correlation (middle), and flow-area (right) methods on the patients' cohort. The transit-time and cross-correlation methods result in better agreements than the flow-area method.

**Figure 6:**
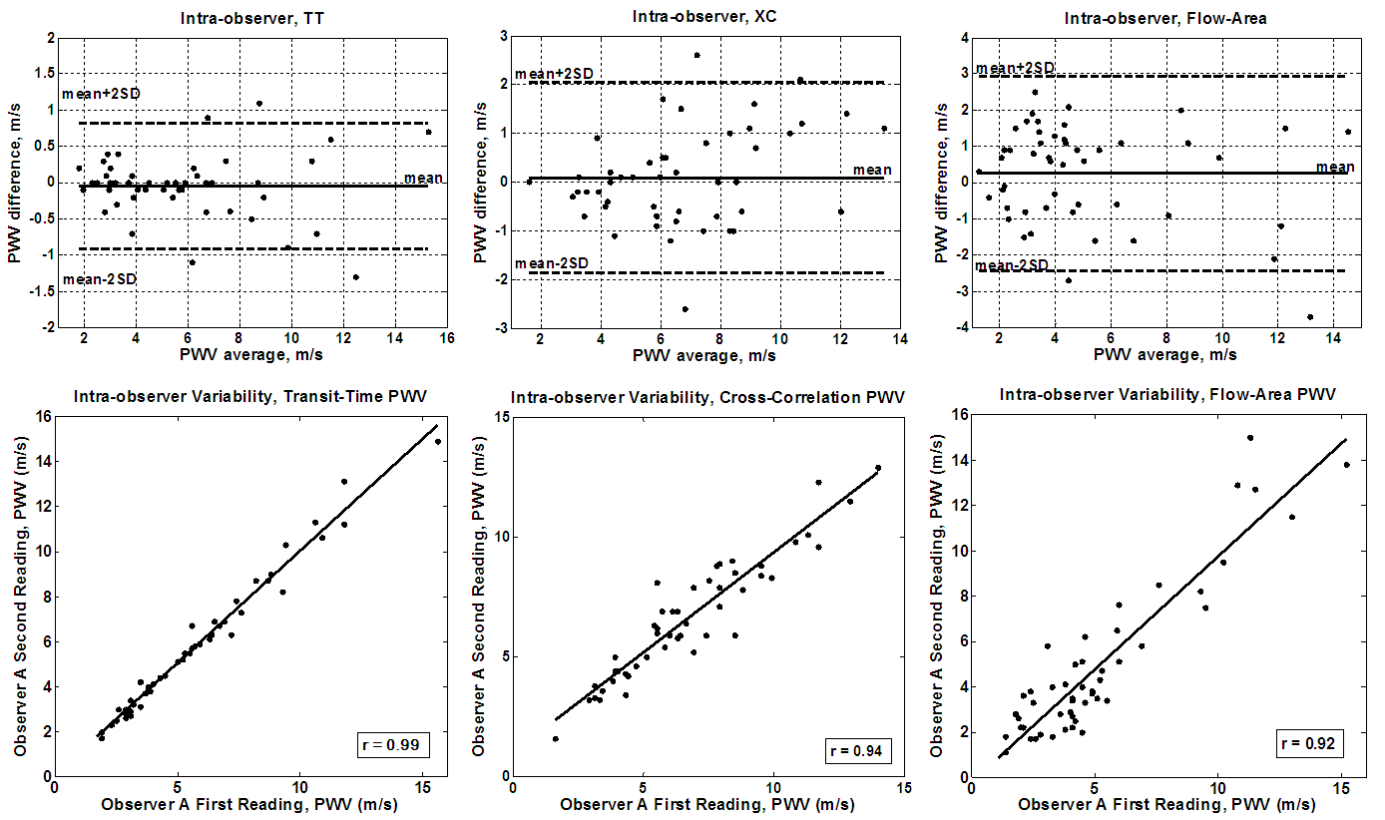
**Intra-observer variability**. Bland-Altman (up) and regression analysis (down) are shown for transit-time (left), cross-correlation (middle), and flow-area (right) methods on the patients' cohort. The transit-time and cross-correlation methods result in better agreements than the flow-area method.

**Figure 7:**
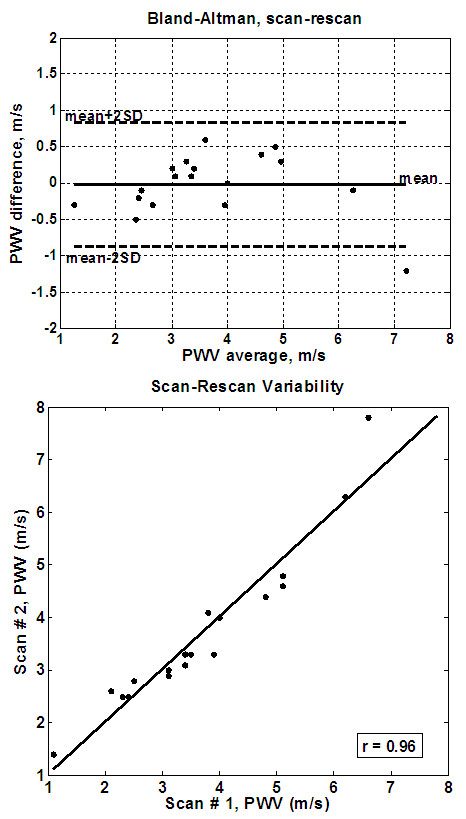
**Inter-scan variability**. Bland-Altman (up) and regression analysis (down) are shown on the volunteers' cohort. There is large agreement between the repeated scans.

Measured PWV values ranged from 1 m/s to 16 m/s, and showed good correlation with patient age (r = 0.81). The average processing times were 23 s, 31 s, and 110 s for the TT, XC, and QA methods, respectively. The Bland-Altman plots for inter-observer variabilities (Figure 5) showed no bias between the two observers using the TT or XC methods. All differences lied within the ± 2SD limit (mean (SD) PWV differences = -0.12 (1.3) m/s and 0.2 (1.3) m/s for the TT and XC methods, respectively). The QA method resulted in larger differences between the two observers (mean (SD) PWV difference = 0.6 (1.6) m/s). The correlation coefficients between the two observers (Figure 5) confirmed the Bland-Altman analysis: r = 0.94 (y = 1.06x - 0.3), 0.88 (y = 0.91x + 0.4), and 0.83 (y = 0.9x - 0.2) for the TT, XC, and QA methods, respectively. Figure 6 shows the results of the intra-observer analysis. The Bland-Altman mean (SD) PWV differences were -0.04 (0.4) m/s, 0.09 (0.9) m/s, and 0.2 (1.4) m/s, and the correlation coefficients were 0.99 (y = 0.99x + 0.067), 0.94 (y = 0.84x + 1.0), and 0.92 (y = 0.99x - 0.23) for the TT, XC, and QA methods, respectively. The results showed no bias between the repeated measurements for all three methods. Figure 7 shows the results of the inter-scan reproducibility (for all three methods). Repeating the scan did not have large effect on the results (r = 0.96 (y=x+0.01) and mean (SD) PWV difference = -0.02 (0.4) m/s).

**Figure 8:**
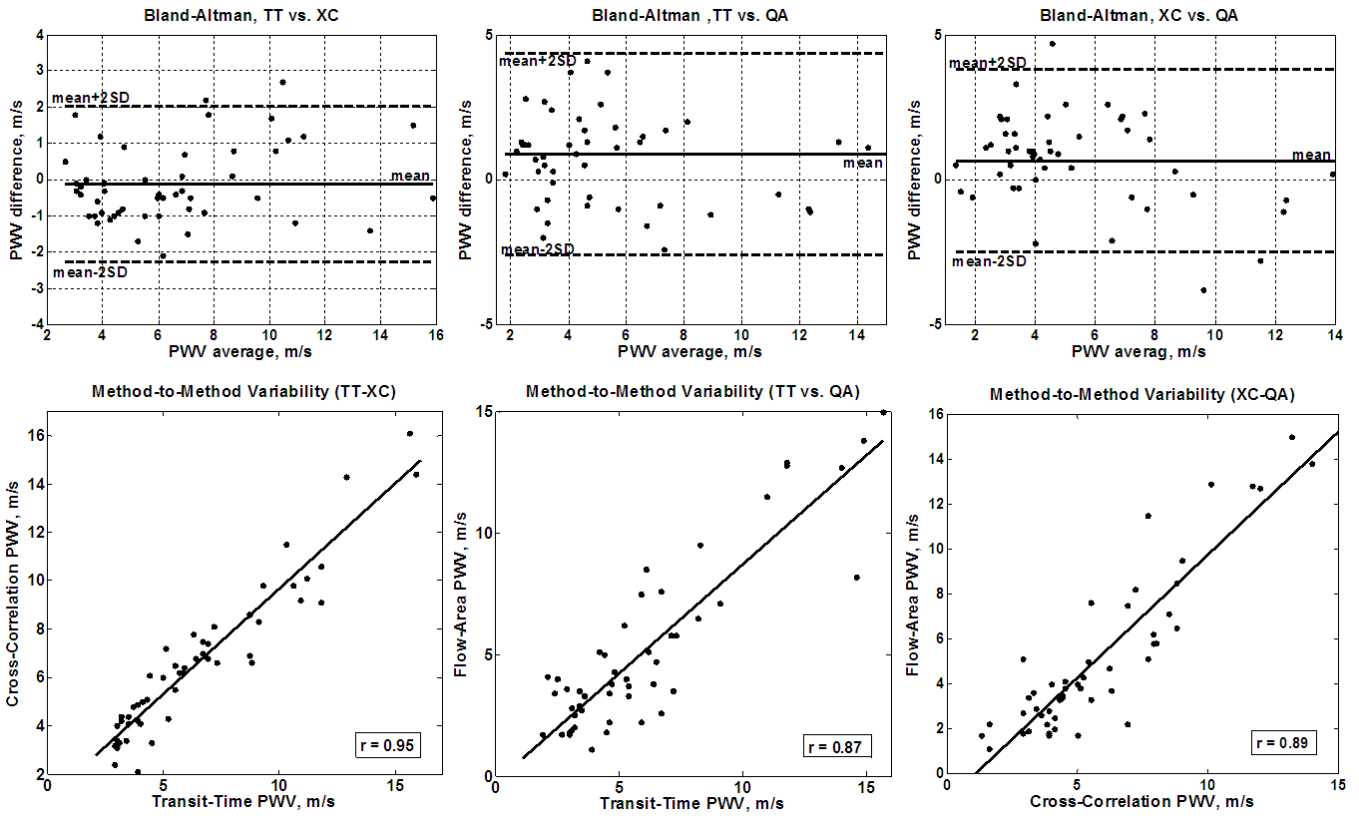
**Inter-method variability**. Bland-Altman (up) and regression analysis (down) are shown for transit-time vs. cross-correlation (left), transit-time vs. flow-area (middle), and cross-correlation vs. flow-area (right) methods on the patients' cohort. The transit-time and cross-correlation methods have large agreement together than with the flow-area method.

Figure 8 shows the regression analysis and Bland-Altman plots for the correlation between different techniques in estimating PWV (TT-XC, TT-QA, and XC-QA). The results showed strong correlation between TT and XC measurements (r = 0.95 (y = 0.87x + 0.95) and mean (SD) PWV difference = -0.12 (1.0) m/s). However, the TT and XC measurements showed less correlation with QA measurements (r = 0.87 (y = 0.89x - 0.28) and 0.89 (y = 1.1x - 1.2), and mean (SD) PWV differences = 0.8 (1.7) m/s and 0.65 (1.6) m/s for TT-QA and XC-QA, respectively). No significant differences were found between the different methods (P > 0.05).

Despite the lack of gold standard catheter measurements in this study, the measured PWV showed good correlation with pulse pressure (r = 0.73) and mvr (r = 0.69) in the group of ischemic patients (n = 25). The mean PWV in this group was 6.7 m/s. The subgroup of patients with PWV above the mean value (n = 11) had average PWV, pulse pressure, and mvr significantly (P < 0.02) larger than those with PWV below the mean value: 4.9 m/s, 70 mmHg, and 1.1 versus 9 m/s, 55 mmHg, and 0.9, respectively.

**Figure 9:**
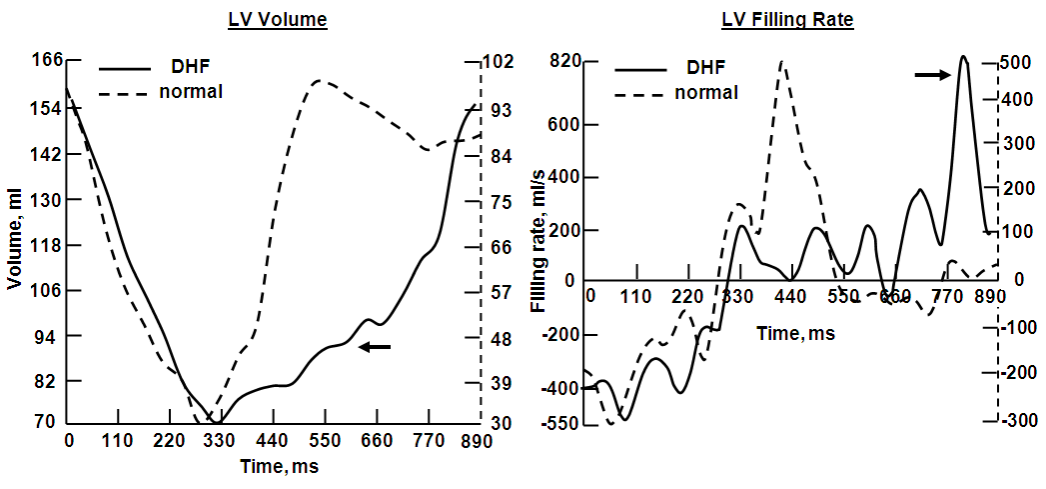
**LV volume and filling rate in diastolic heart failure**. LV volume (left) and filling rate (right) for a diastolic heart failure patient (solid) and a healthy volunteer (dashed). Diastolic heart failure is characterized by a major filling component at the late atrial phase compared to early filling phase (arrows).

**Figure 10:**
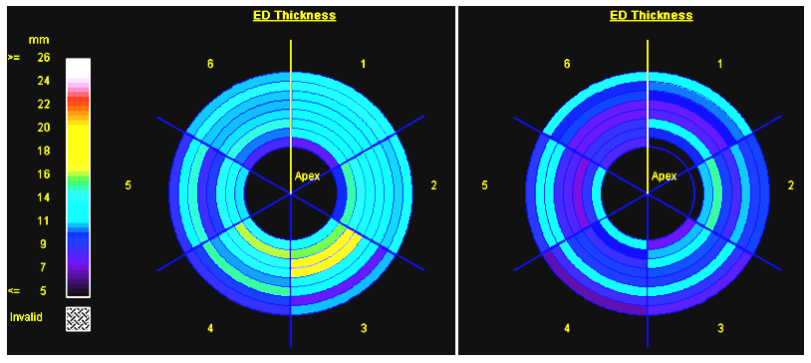
**LV end-diastolic thickness in diastolic heart failure**. Bull's eye figures of end-diastolic thickness in diastolic heart failure (left) and normal (right) computed from a stack of cine short-axis images.

**Figure 11:**
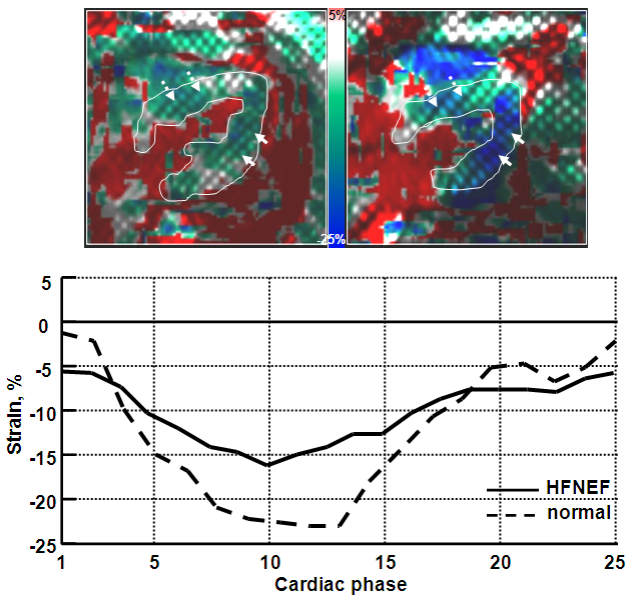
**LV myocardial strain in diastolic heart failure**. The left and right images show (longitudinal) myocardial strains overlaid on four-chamber tagged images at end-systole for diastolic heart failure and normal subjects, respectively. LV is manually traced for clarity. Solid and dashed arrows point to LV free wall and septum, respectively. The curves show longitudinal strain values through the cardiac cycle for patients (solid) and normals (dashed). Diastolic heart failure is characterized by a small strain dynamic range (difference between end-systolic and end-diastolic strains) and less relaxation during diastole, compared to normal.

The results from the diastolic heart failure patients showed the LV filling mechanism to be significantly different compared to volunteers (Figure 9). The patients' flow curves showed diastolic dysfunction, where a major part of LV filling occurred later during the atrial filling phase, despite differences in heart rate. LV relaxation was impaired during most of the diastolic phase. The early/atrial (E/A) flow ratio was different between diastolic heart failure and volunteers (P < 0.01). E/A was less than 1 in the patients (0.54 ± 0.18), and greater than 1 in volunteers (2.1 ± 0.5). LV end-diastolic thickness was larger (P < 0.01) in patients (14.3 ± 3.1 mm) than in volunteers (10.5 ± 2.6 mm), as shown in Figure 10. LV myocardial dynamic strain range (difference between end-systolic and end-diastolic strains) was reduced in the patients (P < 0.005), with less relaxation during the diastolic phase (Figure 11). Strain (longitudinal) ranges were 16.8 ± 2.7 mm^-1 ^and 22.9 ± 3.1 mm^-1 ^in patients and volunteers, respectively. PWV was higher (P < 0.005) in patients (6.3 ± 1.9 m/s) than in volunteers (3.5 ± 1.4 m/s). The degree of LV diastolic dysfunction showed strong correlation with aortic PWV in diastolic heart failure. The correlation coefficients (r) between E/A ratio, myocardial thickness, dynamic strain range and between PWV were -0.83, 0.81, and -0.78, respectively.

## Discussion

CMR provides non-invasive means for estimating systemic compliance by measuring the aortic PWV. TT, XC, and QA are common methods for measuring PWV from velocity-encoded CMR. Each method has its own advantages and disadvantages. The purpose of this study was to objectively compare these methods with each other and to test inter-observer, intra-observer, and inter-scan variabilities. It should be noted that this study did not focus on analyzing the relationship between PWV measurements and different physiological parameters as has been conducted in previous literature [35-41]. Rather, we were interested in studying the reproducibility and repeatability of the available CMR techniques.

Although the current study did not focus on PWV comparison between 1.5-Tesla and 3 Tesla systems, the use of a 3 Tesla system in this study allowed for achieving higher temporal or spatial resolutions, compared to previous results on 1.5-Tesla [9,42,43], which is reflected in the overall reduced variabilities reported in the Results section. Theoretically, high temporal resolution allows for more precise measurement of the wave travelling time (Δt) in the TT and XC methods, which results in more accurate estimation of PWV. Likewise, high spatial resolution allows for more exact measurement of the vessel cross-sectional area in the QA method with more accurate PWV values. Nevertheless, such advantages are usually accompanied with some difficulties, e.g. susceptibility artifacts, which were not a major concern in this study. Other techniques could be implemented to increase the temporal resolution. One possibility is to conduct two consecutive scans with the trigger delay set to TR/2 in the second scan. Interleaving the frames from the two scans would double the temporal resolution. One problem is that this method requires the two scans to have the same chest position and length of the cardiac cycle. Other possibilities can be achieved through pulse sequence optimization by using shorter RF pulses, specialized CMR coils, or faster (steeper) gradients.

The TT method resulted in the most reproducible measurements and required the shortest processing time, followed by the XC method, and then the QA method. The lower reproducibility of the QA method could be attributed to tilted plane orientation in the case of a curved aortic path, or to the manual determination of the vessel boundary in the analysis step. More automated techniques for extracting the vessel cross section should result in more reproducible results. The measurements from the TT and XC methods were closer to each other than to those from the QA method. It should be noted that although the two acquisitions in the TT method provide more data than in the XC or QA methods, this may result in discrepancies related to physiological variations between the two separate slice scans. The XC method required high temporal resolution, while the QA method required high spatial resolution. Both the XC and QA methods required one set of velocity-encoded images, while the TT method required two sets. As the QA method uses only one set of cross-sectional images, this makes it suitable for curved arterial paths, like the aortic arch, and provides the exact PWV at the specified site. On the other hand, the TT and XC methods provide an approximation of the PWV over the length of the vessel. Nevertheless, in the QA method, PWV could have been measured at two sites of the aorta (proximal and distal) and an average is computed. However, this approach doubles the image processing time, which is already long in QA, rendering the technique impractical for this application. For large arteries, like the aorta, the three methods should result in similar measurements, as altered vessel mechanical properties tend to be more systematic [2]. The TT method was the least dependent on user interaction, followed by the XC method, and then the QA method. It should be noted that the points selected along the aortic path in the XC method should not be too close to each other unless very high temporal resolution is achieved. The introduction of very close points tended to lower the estimated PWV. Due to its dependence on user interaction, the QA method resulted in the least reproducible measurements and the largest inter- and intra-observer variabilities. Semi-automated methods could be implemented for segmenting the aortic cross section in the QA method using pixel intensity threshold, e.g. include all pixels within ± 2SD of the blood mean signal intensity. However, there is a problem of possible anatomy overlap with adjacent structures of similar signal intensity. Furthermore, the amount of time needed to review and correct the automatically-selected boundaries can exceed the time required to identify the vessel boundary in all 10-15 frames during early systole.

In this work, velocity was computed as the averaged value across a 5 × 5-pixels ROI centered at the vessel center. It should be noted that other investigators have previously used the region of peak velocity for velocity measurement [44]. The measurements could be different in the two methods, especially in the proximal slice where peak velocity is offset from the vessel center. This may explain the difference in the systolic pulse shape between the proximal and distal curves in the TT method in Figure 1 (it appears more rounded in the proximal slice). Another observation is the difference in the base velocity between the two sites, which could be attributed to local variations in the vessel geometry, vessel curvature, or to reflection waves. It should be also noted that both cross-sectional slices were acquired with one isocenter position (the scanner table did not move between the two acquisitions), and that physiological variations between different breath-holds were not corrected for, which may explain shape variation between the two velocity curves.

The choice of the analysis technique should generally depend on the geometry of the vessel segment at which PWV is measured, and on the available image quality. If possible, images with high temporal (for TT and XC methods) and spatial (for QA method) resolutions should be obtained, to have flexibility in choosing any of the three analysis techniques. The analysis should be repeated in case the calculated PWV is significantly different from expected measurements available in the literature, which could be due to venc aliasing, patient motion, or image artifacts. Despite the wide range of the measured PWV values, the values at the lower and upper ends of the range were similar between the different analysis techniques, which suggests their correctness. The wide range of the measured values could in part be attributed to the heterogeneous cohort of the patients included in the study. More studies are needed to investigate other possible reasons of the wide range of PWV measurements.

Finally, the study gives an example of the importance of measuring PWV in diastolic heart failure. The results provided evidence of both aortic and LV stiffening in diastolic heart failure. Such evidence consisted of slow early filling, increased atrial filling, increased myocardial mass and stiffness, and reduced aortic distensibility. Thus, the results suggests that aortic stiffness is a major input in diastolic heart failure, and it has to be considered when studying different treatment options and for patient follow-up.

Future studies will include the implementation and comparison of the proposed techniques for measuring PWV in vessels other than the aorta (e.g. pulmonary arteries). Imaging protocols with higher temporal and spatial resolutions will be necessary to overcome the problems of pulmonary artery short length and reflection waves [45].

### Limitations

There are a few limitations that must be acknowledged regarding the implemented techniques. Firstly, no invasive catheter measurements, which are considered the gold standard, were acquired in this study. However, PWV has previously been validated against pressure catheters [19-24]. Furthermore, PWV measurements showed good correlations with different cardiac parameters as shown in the Results section. Secondly, the conducted PWV scans were added to the patients' main CMR study. Thus, PWV may have been slightly affected by patient stress when the measurement took place towards the end of the exam. Nevertheless, the volunteer scans showed no systematic difference between the first and second measurements. The third limitation is that the original XC method, as described in [26], requires two CMR acquisitions with orthogonal venc directions (head-to-foot and left-to-right), and it then calculates the net blood velocity by vector summation of the two measurements allowing for analysis of PWV throughout both the transverse and descending aorta. Only the craniocaudal/head-to-foot velocity-encoded images were acquired in our study, allowing acquisition and processing times to be decreased, but limiting our analysis to the descending aorta. Another limitation is that the QA method holds true under the assumption that there are no reflection waves superimposed on the forward-transmitting pressure pulse during early systole, such that the incremental variation in blood flow is proportional to the change in vessel cross-sectional area. This condition was satisfied in all cases in this study; however, reflection waves could be a critical problem in shorter vessels, such as the pulmonary arteries. Another point to be mentioned is that the imaging pulse sequences were not optimized for each measurement method (TT, XC, or QA), e.g. TR minimization for the TT or XC methods. However, this strategy would have resulted in different separate acquisitions and hindered method comparison. Finally, despite the good results from the inter-scan variability test, it should be noted that this study did not account for possible physiological variations between repeated sessions.

## Conclusions

In conclusion, with the current 3-Tesla CMR protocol and analysis methods, the TT and XC methods result in closer and more reproducible aortic PWV measurements, and require less user interaction, than the QA method.

## Competing interests

The authors declare that they have no competing interests.

## Authors' contributions

EHI prepared the imaging protocol, supervised the experiments, analyzed the results, and wrote the manuscript. KRJ analyzed the results for the inter-observer study and helped in editing the manuscript. ABM recruited the diastolic heart failure patients and helped in analyzing their results. JMS conducted the CMR scans. RDW supervised the CMR scans, contributed in interpreting the results, and helped in drafting and reviewing the manuscript.
